# The Effects of Different Exercises on Insulin Resistance and Testosterone Changes in Women with Polycystic Ovarian Syndrome: A Network Meta-Analysis Study

**DOI:** 10.3390/healthcare13172132

**Published:** 2025-08-27

**Authors:** Yuandan Tan, Yujie Liu, Ami Koga, Yuling Yuan, Haohan Yu, Jingmin Liu

**Affiliations:** 1 Division of Sports Science and Physical Education, Tsinghua University, Beijing 100084, China; tyd24@mails.tsinghua.edu.cn (Y.T.); liuyujie24@mails.tsinghua.edu.cn (Y.L.); amygaojpcn@yahoo.co.jp (A.K.); vastime@outlook.com (H.Y.); 2 School of Physical Education, Nanchang University, Nanchang 330031, China; yuanyuling@ncu.edu.cn

**Keywords:** polycystic ovary syndrome, exercise therapy, insulin resistance, testosterone, network meta-analysis

## Abstract

Objective: To compare the efficacy of exercise modalities for simultaneously improving homeostasis model assessment for insulin resistance (HOMA-IR) and total testosterone in women with polycystic ovary syndrome (PCOS). Methods: We conducted a Bayesian network meta-analysis of 19 randomized controlled trials (n = 808) to evaluate six exercise interventions: yoga, moderate-intensity continuous training (MICT), high-intensity interval training (HIIT), resistance training (RT), combined aerobic-resistance training (CT), and control (CG). Primary outcomes were changes in HOMA-IR and total testosterone, with interventions ranked via surface under the cumulative ranking curve (SUCRA). Results: For HOMA-IR reduction, yoga (SUCRA = 90.73%; SMD = −0.73, 95% CrI: −1.3 to −0.086) and HIIT (SUCRA = 74.12%; SMD = −0.47, 95% CrI: −0.75 to −0.15) demonstrated superior efficacy versus MICT (SUCRA = 50.56%) and CT (SUCRA = 42.29%), while RT was the least effective (SUCRA = 32.53%). For testosterone lowering, yoga was ranked the highest again (SUCRA = 92.46%; SMD = −0.85, 95% CrI: −1.7 to −0.12), followed by MICT (SUCRA = 75.72%; SMD = −0.56, 95% CrI: −0.97 to −0.25) and HIIT (SUCRA = 61.12%; SMD = −0.42, CrI: −0.88 to −0.12). CT and RT showed non-significant effects for both outcomes (*p* > 0.05). Conclusions: Yoga is the optimal intervention for dual-pathway improvement in PCOS. HIIT and MICT provide outcome-specific benefits (metabolic vs. endocrine), whereas CT and RT necessitate protocol refinement. Systematic review registration: This systematic review and network meta-analysis study was registered in PROSPERO (CRD420251011979).

## 1. Introduction

Polycystic ovary syndrome (PCOS), a common endocrine condition, impacts an estimated 4–21% of reproductive-aged women worldwide [1]. It is characterized by ovulatory dysfunction, hyperandrogenism, and polycystic ovarian morphology [2]. The pathogenesis of PCOS is not fully understood, but it may be associated with hyperandrogenism, insulin resistance (IR), obesity, metabolic abnormalities, and inflammation [3]. PCOS is underpinned by insulin resistance and compensatory hyperinsulinemia in 60–80% of affected individuals [4], a metabolic core that drives both reproductive dysfunction and increased cardiometabolic risk. This dual metabolic–endocrine pathology manifests in metabolic abnormalities: it exacerbates ovarian androgen overproduction and reduces sex hormone-binding globulin (SHBG) synthesis [5], amplifying hyperandrogenic reproductive manifestations such as hirsutism, acne, and anovulation [6]. Beyond reproductive sequelae, this pathology extends to cardiovascular risks, including heightened susceptibility to type 2 diabetes, gestational diabetes [7], atherogenic dyslipidemia [8], and heightened risks of coronary heart disease [9] and cerebrovascular events [10]. While exercise is a cornerstone of PCOS management [11,12], critical uncertainty remains regarding which exercise modalities most effectively improve both metabolic (IR) and hormonal (hyperandrogenism) outcomes simultaneously. Given the central role of IR and hyperandrogenism in PCOS pathophysiology, this NMA prioritized HOMA-IR and total testosterone as co-primary outcomes to evaluate exercise efficacy against core disease mechanisms.

Current management of PCOS is individualized and often requires a combination of lifestyle modifications and pharmacotherapy [13]. Lifestyle modification, which encompasses diet, exercise, and behavioral interventions, constitutes first-line therapy in international guidelines [11,12]. Exercise specifically improves insulin sensitivity and reduces hyperandrogenemia through mechanisms including enhanced glucose disposal, SHBG upregulation, and visceral adiposity reduction [14]. Meta-analyses confirm structured exercise reduces homeostasis model assessment of insulin resistance (HOMA-IR) and free androgen index (FAI) in PCOS [15].

Despite the growing body of research on exercise interventions for PCOS, several critical evidence gaps persist in the current literature. Previous studies have yielded inconsistent findings regarding the optimal exercise modalities for PCOS management. For instance, some studies indicate that aerobic training is more effective in reducing fasting insulin levels [16], while another meta-analysis of RCTs indicates that aerobic and resistance training demonstrates benefits for cardiorespiratory fitness and waist circumference reduction in PCOS patients, it reports minimal impact on fasting glucose, insulin resistance markers, or reproductive hormone profiles [17]. High-intensity interval training (HIIT) has shown superior improvements in HOMA-IR compared to moderate continuous training (MICT) in certain trials [18], but this finding is not consistent across all studies [14], likely due to variations in training intensity, intervention duration, and participant baseline BMI across studies. Furthermore, conventional meta-analyses cannot hierarchically rank exercise modalities for their ability to improve both IR and hyperandrogenism simultaneously, as they lack the methodology to integrate direct and indirect comparative evidence across heterogeneous interventions [19]. From a clinical perspective, prior syntheses have not evaluated IR (HOMA-IR) and hyperandrogenism (testosterone) as co-primary outcomes, despite these being key pathophysiological drivers of PCOS-related morbidity.

Network meta-analysis (NMA) offers a robust methodological framework that allows for the simultaneous comparison of multiple interventions by integrating direct and indirect evidence across a network of randomized controlled trials [20]. NMA not only provides estimates of relative effectiveness among treatments that have not been directly compared but also ranks interventions based on their efficacy and safety profiles [21]. Specifically, we employed the surface under the cumulative ranking curve (SUCRA) method to quantify the probability of each exercise modality being the most effective, thereby providing a clear hierarchy of interventions beyond simple mean difference comparisons. NMA overcomes this limitation by integrating direct and indirect evidence across trials, enabling us to rank exercise modalities by their efficacy in improving both HOMA-IR and total testosterone, the key outcomes central to PCOS pathophysiology. This NMA adheres to the Preferred Reporting Items for Systematic Reviews and Meta-Analyses (PRISMA) guidelines and allows both direct and indirect comparisons across different exercise regimens. These findings will provide clinicians with evidence-based guidance to tailor exercise prescriptions, targeting both metabolic and endocrine dysfunction in PCOS to optimize patient outcomes.

## 2. Materials and Methods

This NMA adhered to the PRISMA guidelines [22]. Table S1 shown the PRISMA for network meta-analysis checklist. A predefined protocol was registered prospectively with the International Prospective Register of Systematic Reviews (PROSPERO) on 16 March 2025 (registration number: CRD420251011979).

### 2.1. Database Search and Identification

A systematic search of PubMed, EBSCO, Embase, Cochrane CENTRAL, and Web of Science (inception to March 2025) identified RCTs comparing exercise interventions in PCOS. We used MeSH terms, Embase, free text methods, and expert opinion to identify all relevant search terms related to exercise, physical activity, and PCOS. The detailed search strategies are provided in Table S2. We also reviewed all existing systematic reviews and meta-analyses to ensure that all appropriate references were included in this process.

### 2.2. Inclusion and Exclusion Criteria

Studies were included based on the PICOS criteria. The population of interest were reproductive-aged women (aged 15 years and above) diagnosed with PCOS according to the Rotterdam or NIH criteria. The intervention comprised structured exercise programs with a duration of at least four weeks and the comparator was either a non-exercise control group or an alternative exercise modality. Studies involving structured dietary co-interventions were excluded to eliminate confounding and precisely attribute outcomes to exercise modalities per our causal isolation objective. The primary outcomes measured were changes in HOMA-IR and total testosterone (mean change ± SD). Only RCTs were considered for inclusion in the study. Trials were excluded if they involved pharmacological co-interventions (unless these had been stable for at least three months), had fewer than 7 participants per arm, or were only available as abstracts without full data.

### 2.3. Data Extraction

Data extraction encompassed participant demographics, study design characteristics, detailed exercise intervention parameters, and all relevant outcomes. Where critical data elements were missing from published reports, we proactively contacted the corresponding authors for clarification. To maintain data integrity, any discrepancies identified during extraction were meticulously resolved through iterative discussion among reviewers to reach consensus; unresolved issues were adjudicated by a third reviewer. This rigorous process was implemented to guarantee consistent and accurate data handling, thereby strengthening the reliability and validity of our network meta-analysis results.

### 2.4. Risk of Bias Assessment

The Cochrane recommended risk assessment tool RevMan5.4 was used to assess the quality of the included studies, including random sequence generation, assignment concealment, blinding, incomplete outcome data, selective reporting, and other biases. The literature quality was divided into three levels from high to low: low risk, medium risk, and high risk. If the above quality evaluation criteria are fully met, the study has a low-risk level and the possibility of bias is small. If the above quality evaluation criteria are partially met, the risk is medium and the possibility of bias is moderate. If the above quality evaluation criteria are not met at all, the study is high risk and the possibility of bias is high [23].

### 2.5. Statistical Analysis

A frequentist random-effects NMA was conducted using the R package netmeta (version 4.5.0) to synthesize direct and indirect evidence across exercise interventions [24]. The heterogeneity variance was estimated via the DerSimonian–Laird method [25]. Standardized mean differences (SMDs) with 95% confidence intervals (CIs) were calculated for changes in HOMA-IR and total testosterone using Cohen’s method. In terms of data extraction, for studies reporting mean change ± SD, values were directly extracted. When only pre-/post-intervention means ± SDs were available, we applied methods from the Cochrane Handbook to compute mean change and SDs of change [26]. Standard errors (SEs) were converted to SDs using the formula SD = SE× n. For studies reporting medians and interquartile ranges (IQRs), means and SDs were estimated using established methods [27,28]. If SDs/SEs were unreported, pooled SDs from other included trials in the same outcome analysis were imputed [27,29].

The statistical analyses were executed utilizing the netmeta package [30], implemented within an online R-based platform specifically designed for network meta-analysis. First, we constructed a network diagram to visualize all exercise interventions. Bayesian NMAs were performed via the MetaInsight tool (version V4.5.0), running Markov chain Monte Carlo simulations with four chains and a total of 70,000 iterations (burn-in period of 20,000). Convergence of the model was tested via the Gelman–Rubin convergence assessment. Based on pre-established interstudy heterogeneity, random-effects analyses of WMD were selected. Inconsistency between direct and indirect effect size comparisons were assessed via node-splitting models [31] with corresponding Bayesian *p*-values. A *p*-value exceeding 0.05 indicates that there is acceptable consistency between direct and indirect evidence estimates at that node. The comparative effectiveness ranking of all interventions was derived from the SUCRA values. SUCRA values, which estimate the probability of an intervention being optimal (range: 0–100%), were used to rank efficacy. Higher SUCRA percentages are indicative of a greater likelihood of an intervention being the most effective option [32].

### 2.6. Sensitivity Analysis

To evaluate the robustness of our primary findings, we conducted sensitivity analyses. Each trial was individually omitted and the network was refitted. Resulting estimates were then juxtaposed with the primary model both visually and quantitatively to verify that no single study exerted undue leverage on the overall findings or materially altered the direction of treatment effects.

### 2.7. Publication Bias

Potential publication bias was initially assessed through visual inspection of funnel plots. Statistical evaluation was subsequently performed using Egger’s regression test [33]. An Egger’s test *p*-value > 0.05 was interpreted as indicating no substantial evidence of publication bias within the analyzed network.

## 3. Results

### 3.1. Study Identification and Network Model Construction

**Figure 1:**
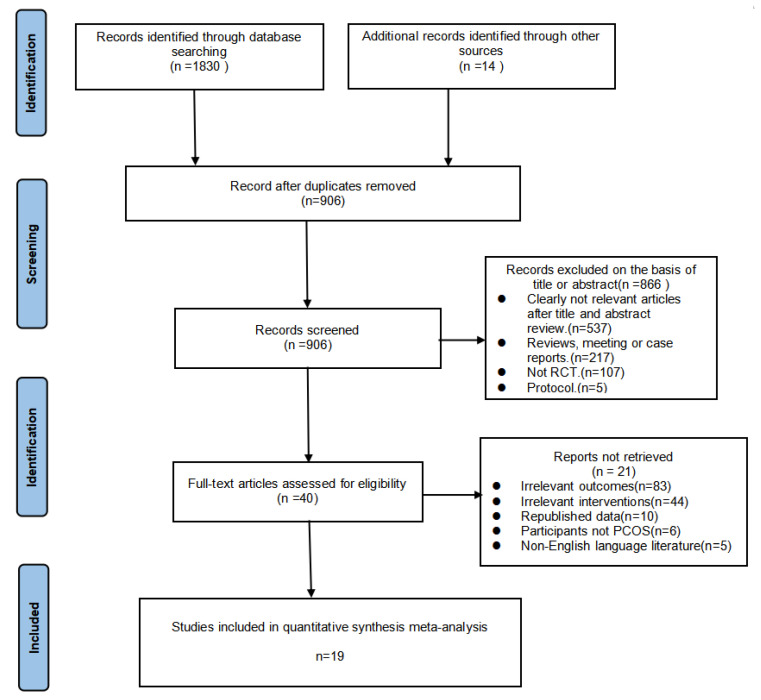
PRISMA systematic review and meta-analysis flow chart. Note: A list of studies excluded after full-text assessment with reasons is available in Supplementary Table S3.

Following the PRISMA guidelines, a total of 1830 records were identified through database searches, with 14 additional trials sourced from reference lists. Following removal of duplicates and sequential screening of titles, 19 randomized controlled trials ultimately satisfied our predefined inclusion criteria [15,34,35,36,37,38,39,40,41,42,43,44,45,46,47,48,49,50,51]. The complete study selection process is detailed in Figure 1

A total of 19 studies involving 891 subjects were included. The included trials were published from 2008 to 2025. Participants’ mean age ranged from 16 to 32 years. Intervention duration ranged between 8 and 24 weeks. Of 19 trials, the most common intervention was moderate-intensity aerobic training which has 12 trials (MICT), followed by high-intensity interval training which has 8 trials (HIIT), the intervention program in 3 trials was conducted based on resistance training (RT); 3 trials were based on yoga, and 3 trials were concurrent training (CT). Detailed characteristics of the included studies including publication year, first author, country, sample size, age, sex, diagnostic criteria, treatment and control group (CG) intervention, and treatment period are described in Table S4 [15,34,35,36,37,38,39,40,41,42,43,44,45,46,47,48,49,50,51].

### 3.2. Risk of Bias of the Included Studies

In random sequence generation, two studies were graded as high. In allocation concealment, eight studies were graded as unclear and two studies were graded as high. A toral of 12 studies were graded as unclear and five studies were graded as high in the blinding of participants. Furthermore, four studies were graded as unclear. In the blinding of outcome assessment, 13 studies were graded as unclear. In incomplete outcome data, all studies showed low risk of bias. Regarding selective outcome reporting, all included studies comprehensively reported their predefined primary and secondary outcomes, leading to a unanimous low risk of bias assessment for this domain. Other sources of bias were unclear; all studies were graded as unclear. Detailed visualization of each study with the risk of bias graph is presented in Figures S1 and S2.

### 3.3. Network Meta-Analysis

#### 3.3.1. Evidence Network

**Figure 2:**
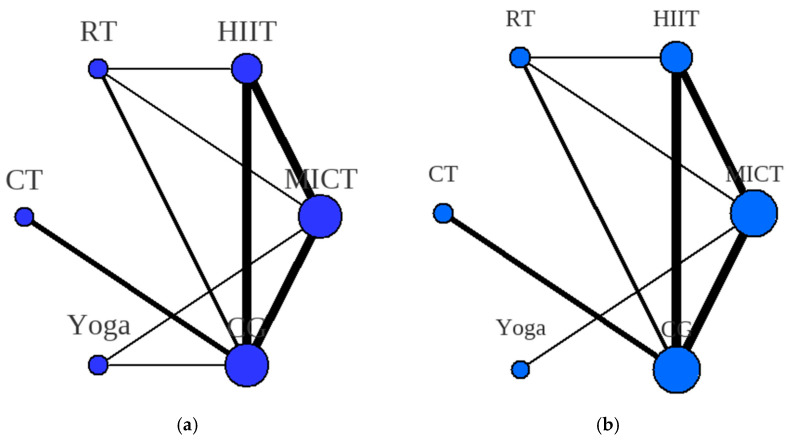
(**a**) HOMA-IR network meta-analysis maps, (**b**) total testosterone network meta-analysis maps. Note: MICT = moderate-intensity continuous aerobic training; HIIT = high-intensity interval training; RT = resistance training; CT = concurrent training; Yoga = yoga; CG = control group.

Network plots visually represent the available evidence for comparing interventions. Nodes correspond to the evaluated interventions, and the connecting lines between nodes signify the presence of direct head-to-head comparisons within the included trials. The size of each node is proportional to the total number of participants randomized to that specific intervention across all studies. The thickness of each connecting line reflects the number of trials directly comparing those two interventions. A total of 16 studies presented data on HOMA-IR, which included six different interventions and 14 studies presented data on total testosterone, which included six different interventions (refer to Figure 2a for HOMA-IR and Figure 2b for total testosterone).

#### 3.3.2. Effect on HOMA-IR in PCOS

**Figure 3:**
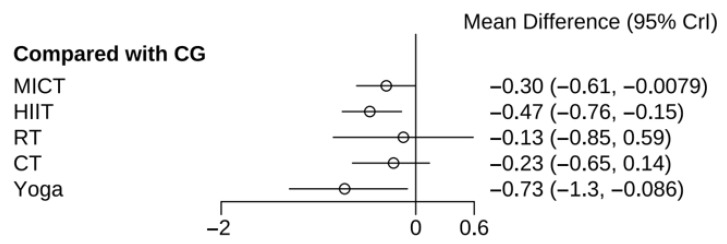
Results of various exercises compared with CG in improving HOMA-IR in women with PCOS.

Sixteen studies (n = 696) provided data on HOMA-IR changes. Pairwise comparisons revealed statistically significant reductions in HOMA-IR for yoga, MICT, and HIIT compared to the CG. Other comparisons between active interventions did not reach statistical significance (Figure 3). To comprehensively rank the interventions based on their probability of being optimal for HOMA-IR reduction, we computed the SUCRA values, the specific results are shown in Table 1.

**Figure 4:**
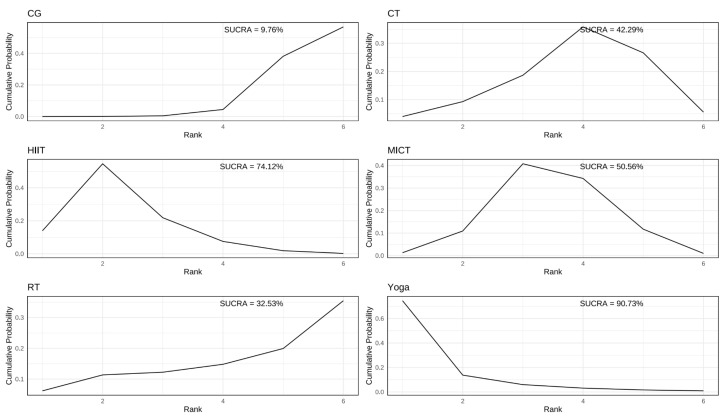
SUCRA rankogram plots on HOMA-IR in women with PCOS.

Based on the SUCRA analysis, yoga demonstrated (Figure 4 and Figure S3) the highest probability of being the most effective intervention for improving IR (SUCRA = 90.73%; SMD = −0.73, 95% CrI: −1.3 to −0.086), HIIT ranked second (SUCRA = 74.12%; SMD = −0.47, 95% CrI: −0.75 to −0.15). MICT (SUCRA = 50.56%; SMD = −0.3, CrI: −0.61 to −0.01) and CT (SUCRA = 42.29%; SMD = −0.23, CrI: −0.65 to 0.14) showed modest effects. RT was the least effective (SUCRA = 32.53%; SMD = −0.13, 95% CrI: −0.85 to 0.59).

#### 3.3.3. Effect on Total Testosterone in PCOS

**Figure 5:**
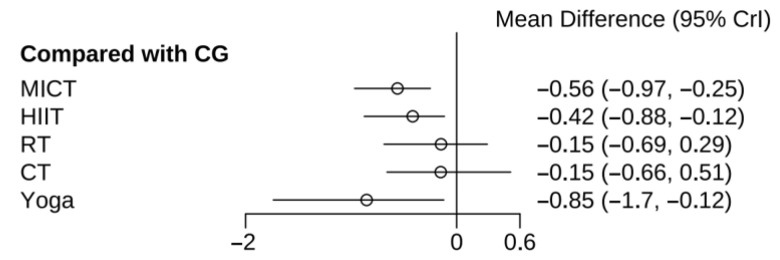
Results of various exercises compared with CG at reducing total testosterone in women with PCOS.

In the study of the effects of different exercises on total testosterone in PCOS patients, a total of 14 studies reported total testosterone levels in 710 subjects. The results indicated that yoga, MICT, and HIIT all produced statistically significant reductions in total testosterone relative to the CG. Differences among other active interventions were not statistically significant (Figure 5).

SUCRA ranking positioned yoga as the intervention with the highest probability of reduction in total testosterone (Table 2).

**Figure 6:**
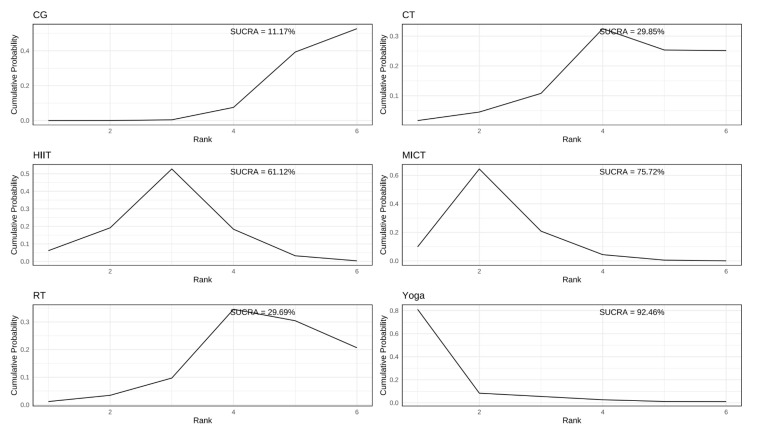
SUCRA rankogram plots on total testosterone in women with PCOS.

Consistent with its top rank position (Figure 6 and Figure S4), yoga exhibited the highest SUCRA value for lowering total testosterone (SUCRA = 92.46%; SMD = −0.85, 95% CrI: −1.7 to −0.12), MICT ranked second (SUCRA = 75.72%; SMD = −0.56, 95% CrI: −0.97 to −0.25). HIIT (SUCRA = 61.12%; SMD = −0.42, CrI: −0.88 to −0.12) and CT (SUCRA = 29.85%; SMD = −0.15, CrI: −0. 66 to 0.51) showed modest effects. RT was the least effective (SUCRA = 29.69%; SMD = −0.69, 95% CrI: −0.29 to 0.51).

#### 3.3.4. Inconsistency Testing

To assess the consistency between direct and indirect evidence sources within our network meta-analysis models, we employed node-splitting methods. Results for HOMA-IR (Figure S5) and total testosterone (Figure S6) revealed Bayesian *p*-values exceeding 0.05 for all comparisons, indicating no statistically significant inconsistency and supporting the validity of the network meta-analysis approach for synthesizing the evidence.

#### 3.3.5. Sensitivity Analysis Findings

In our study, to ensure the reliability and robustness of our analysis, we took a cautious approach to avoid the impact of potential systematic errors on our findings by removing each study individually. Through this step, we were able to assess and confirm that our core findings remained stable and consistent when individual studies were removed one by one, suggesting that the exclusion of certain data points did not have a significant impact on the reliability of the findings. For more detailed insights, refer to Figures S7 and S8.

#### 3.3.6. Publication Bias Findings

We rigorously evaluated publication bias through funnel plot and Egger’s regression test. The funnel plots (Figures S9 and S10) showed that most dots were symmetrically placed around the vertical line of the comparison-specific pooled effect, showing a consistent effect size distribution across studies. Yet, some asymmetry was evident, hinting at possible publication bias, such as underrepresentation of smaller, less precise studies. To verify this visual analysis, we conducted Egger’s regression test. The resulting *p*-value = 0.0761 exceeding 0.05 supported the funnel plot’s visual interpretation, indicating no significant publication bias in our network meta-analysis data.

## 4. Discussion

This systematic review and Bayesian network meta-analysis evaluated the comparative efficacy of five distinct exercise modalities (MICT, HIIT, RT, CT, yoga) on HOMA-IR and lowering total testosterone levels in women diagnosed with PCOS. The HOMA-IR results showed that yoga is the most effective exercise in women with PCOS, HIIT also provides significant benefits, while MICT shows moderate improvements. RT and CT, although less effective, still offer positive outcomes. In terms of reducing testosterone values, yoga again ranked the highest, with MICT and HIIT also showing significant reductions. RT and CT failed to achieve statistical significance, likely due to limited data and heterogeneity in training protocols. Among them, yoga (SUCRA 90.73% for HOMA-IR; 92.46% for testosterone reduction) disrupts this vicious cycle more effectively than other modalities. This dual efficacy is clinically paramount, as simultaneous targeting of IR and hyperandrogenism may mitigate long-term cardiometabolic risks and anovulatory morbidity in women with PCOS.

This network meta-analysis compared the effects of different exercise modalities on anti-insulin stability and testosterone values in women with infertile PCOS. We included HOMA-IR and total testosterone values as primary endpoints. Fasting glucose, fasting insulin, and free testosterone were also assessed as secondary endpoints. Previously, one large-scale NMA [19] has examined the effects of three exercise training interventions (aerobic exercise, resistance training, and yoga) in patients with polycystic ovary syndrome. They have primarily examined the effects of BMI, serum FSH and LH concentrations, and menstruation. In contrast, we performed analyses of the effects of five exercise modalities (intensity continuous training, high-intensity interval training, resistance training, combined aerobic-resistance training, and yoga) on anti-insulin stability and testosterone values in patients with polycystic ovary syndrome. Furthermore, by distinctly classifying CT and isolating RT, our study offers a more granular assessment of the therapeutic potential across this broader spectrum of physical activity modalities.

The observed differential effects on HOMA-IR and total testosterone likely arise from distinct underlying physiological mechanisms activated by each exercise modality. Consistent with previous studies [52,53], yoga, MICT, and HIIT all exhibited significant facilitative effects on insulin sensitivity (HOMA-IR). Proposed mechanisms for this improvement include enhanced skeletal muscle glucose uptake, improved mitochondrial function, and reduced visceral fat accumulation [54,55]. Notably, yoga consistently emerged as the most effective intervention across both primary outcomes (HOMA-IR and total testosterone reduction). Yoga’s pronounced effect on reducing total testosterone could potentially stem from its modulatory actions on the hypothalamic–pituitary–ovarian (HPO) axis [56]. HIIT’s stronger effect on HOMA-IR may stem from its efficient activation of AMP-activated protein kinase, promoting glucose uptake and lipid oxidation [57]. In contrast, MICT showed greater efficacy in reducing total testosterone levels compared to HIIT. Its gradual intensity might favor sustained hormonal regulation pathways [58,59]. CT yielded modest enhancements in insulin sensitivity [60,61], potentially reflecting a synergy between the glucose disposal mechanisms stimulated by aerobic components and the muscle mass augmentation promoted by resistance elements. However, the wide confidence intervals indicate considerable uncertainty, possibly reflecting limited sample sizes or heterogeneity in training protocols. RT exhibited limited efficacy for HOMA-IR improvement. This suggests that in the context of PCOS, the insulin-sensitizing benefits derived solely from muscle hypertrophy may be inadequate, potentially necessitating the inclusion of aerobic exercise components.

This study has several limitations that must be considered when interpreting the findings. First, significant heterogeneity in exercise prescription parameters (e.g., intensity, duration, frequency) and baseline participant characteristics (e.g., age, BMI, PCOS phenotype) limited our ability to perform subgroup or stratified analyses. The relatively small number of included trials (n = 19) further constrained these explorations. Second, the focus on HOMA-IR and total testosterone, while clinically justified, came at the expense of analyzing other hormonal biomarkers (e.g., LH, FSH, AMH) due to their inconsistent reporting across studies. Additionally, the reliance on single-time-point measurements in the available literature precludes insight into dynamic, exercise-induced endocrine changes. Third, the methodological decision to exclude trials with structured dietary co-interventions was necessary to isolate the effect of exercise but consequently limits the generalizability of our findings to real-world clinical settings where combined lifestyle therapy is the norm. Fourth, the statistical reliability of comparisons for some interventions (e.g., CT vs. RT) is limited by reliance on indirect evidence. Wider confidence intervals for certain modalities (e.g., yoga, CT) also indicate that their ranking should be interpreted with caution. Finally, although Egger’s test did not reach formal statistical significance (*p* = 0.0761), observed funnel plot asymmetry raises the possibility of publication bias, potentially from the underrepresentation of smaller studies with null results. It should be noted, however, that sensitivity analyses confirmed the robustness of the primary outcome estimates.

Future high-quality, multicenter RCTs are essential to address these gaps. Such trials should employ rigorously standardized FITT principles, implement stratified randomization by key baseline characteristics (e.g., BMI, phenotype), and incorporate more comprehensive and dynamic hormonal assessments to better elucidate the underlying mechanisms. Research should also explore the integrated effects of exercise and nutrition to delineate optimal, personalized therapeutic strategies. These efforts will be crucial for advancing PCOS management towards mechanism-driven, precision exercise prescription.

## 5. Conclusions

This network meta-analysis establishes that among five exercise modalities evaluated, yoga was found to be the most effective exercise for improving both insulin resistance and testosterone levels in women with PCOS. HIIT and moderate-intensity training also showed benefits. HIIT works better for lowering insulin and MICT is more effective for reducing testosterone, while CT and RT require further optimization. These findings can help guide personalized exercise recommendations.

## Figures and Tables

**Table 1:** Comparative efficacy of exercise modalities on HOMA-IR in PCOS.

	MICT	HIIT	RT	CT	Yoga	CG
**MICT**	MICT	−0.17 (−0.48, 0.2)	0.18 (−0.55, 0.9)	0.07 (−0.44, 0.55)	−0.43 (−0.95, 0.19)	0.3 (0.01, 0.61)
**HIIT**	0.17 (−0.2, 0.48)	HIIT	0.34 (−0.41, 1.07)	0.24 (−0.31, 0.69)	−0.26 (−0.87, 0.39)	0.47 (0.15, 0.76)
**RT**	−0.18 (−0.9, 0.55)	−0.34 (−1.07, 0.41)	RT	−0.11 (−0.92, 0.7)	−0.6 (−1.49, 0.33)	0.13 (−0.59, 0.85)
**CT**	−0.07 (−0.55, 0.44)	−0.24 (−0.69, 0.31)	0.11 (−0.7, 0.92)	CT	−0.5 (−1.16, 0.29)	0.23 (−0.14, 0.65)
**Yoga**	0.43 (−0.19, 0.95)	0.26 (−0.39, 0.87)	0.6 (−0.33, 1.49)	0.5 (−0.29, 1.16)	Yoga	0.73 (0.09, 1.3)
**CG**	−0.3 (−0.61, −0.01)	−0.47 (−0.76, −0.15)	−0.13 (−0.85, 0.59)	−0.23 (−0.65, 0.14)	−0.73 (−1.3, −0.09)	CG

Note: MICT = moderate-intensity continuous aerobic training; HIIT = high-intensity interval training; RT = resistance training; CT = concurrent training; Yoga = yoga; CG = control group.

**Table 2:** Comparative efficacy of exercise modalities on total testosterone in PCOS.

	MICT	HIIT	RT	CT	Yoga	CG
**MICT**	MICT	0.14 (−0.28, 0.52)	0.41 (−0.12, 0.94)	0.41 (−0.16, 1.23)	−0.3 (−1.03, 0.42)	0.56 (0.25, 0.97)
**HIIT**	−0.14 (−0.52, 0.28)	HIIT	0.27 (−0.26, 0.85)	0.27 (−0.29, 1.14)	−0.44 (−1.25, 0.4)	0.42 (0.12, 0.88)
**RT**	−0.41 (−0.94, 0.12)	−0.27 (−0.85, 0.26)	RT	0 (−0.65, 0.88)	−0.71 (−1.62, 0.18)	0.15 (−0.29, 0.69)
**CT**	−0.41 (−1.23, 0.16)	−0.27 (−1.14, 0.29)	0 (−0.88, 0.65)	CT	−0.7 (−1.84, 0.15)	0.15 (−0.51, 0.66)
**Yoga**	0.3 (−0.42, 1.03)	0.44 (−0.4, 1.25)	0.71 (−0.18, 1.62)	0.7 (−0.15, 1.84)	Yoga	0.85 (0.12, 1.74)
**CG**	−0.56 (−0.97, −0.25)	−0.42 (−0.88, −0.12)	−0.15 (−0.69, 0.29)	−0.15 (−0.66, 0.51)	−0.85 (−1.74, −0.12)	CG

Note: MICT = moderate-intensity continuous aerobic training; HIIT = high-intensity interval training; RT = resistance training; CT = concurrent training; Yoga = yoga; CG = control group.

## Data Availability

Data sharing is not applicable. No new data were created or analyzed in this study.

## Supplementary Materials

The following supporting information can be downloaded at https://www.mdpi.com/article/10.3390/healthcare13172132/s1, Table S1: PRISMA for network meta-analysis checklist; Table S2: Keywords and search results in difference databases; Table S3: Studies excluded from the analysis along with the reasons for their exclusion; Table S4: A list of basic characteristics of the studies included in the meta-analysis; Figure S1: Summary of the quality assessment for included studies; Figure S2: Risk of bias graph; Figure S3: Probability ranking diagram of different exercise interventions aimed at improving HOMA-IR in women with PCOS; Figure S4: Probability ranking diagram of different exercise interventions aimed at reducing total testosterone in women with PCOS; Figure S5: Individual study results (with studies excluded) on HOMA-IR grouped by treatment comparison; Figure S6: Individual study results (with studies excluded) on total testosterone grouped by treatment comparison; Figure S7: The forest plots display the HOMA-IR results of the sensitivity analysis; Figure S8: The forest plots display the total testosterone results of the sensitivity analysis; Figure S9: Publication bias funnel plots on HOMA-IR in women with PCOS; Figure S10: Publication bias funnel plots on total testosterone in women with PCOS.
