# Supplementary material for: The Effects of Different Exercises on Insulin Resistance and Testosterone Changes in Women with Polycystic Ovarian Syndrome: A Network Meta-Analysis Study
Source: Healthcare (Basel). 2025 Aug 27;13(17):2132. doi: 10.3390/healthcare13172132 (PMC12427719; doi:10.3390/healthcare13172132)
Supplement: Supplementary file 1 [file healthcare-13-02132-s001.zip › healthcare-3796063-supplementary.pdf]

Table S1: PRISMA for network meta-analysis checklist

Section and Topic	#	Checklist item	Location
**Title**			
**Title**	1	Identify the report as a systematic review incorporating a network meta-analysis (or related form of meta-analysis).	Title
**Abstract**			
**Structured summary**	2	Provide a structured summary including, as applicable: Background: main objectives / Methods: data sources; study eligibility criteria, participants, and interventions; study appraisal; and synthesis methods, such as network meta-analysis. / Results: number of studies and participants identified; summary estimates with corresponding confidence/credible intervals; treatment rankings may also be discussed. Authors may choose to summarize pairwise comparisons against a chosen treatment included in their analyses for brevity. / Discussion/conclusions: limitations; conclusions and implications of findings. / Other: primary source of funding; systematic review registration number with registry name.	Abstract
**Introduction**			
**Rationale**	3	Describe the rationale for the review in the context of what is already known, including mention of why a network meta-analysis has been conducted.	Introduction / 1st and 2nd paragraph
**Objectives**	4	Provide an explicit statement of questions being addressed, with reference to participants, interventions, comparisons, outcomes, and study design (PICOS).	Introduction / 3rd paragraph
**Methods**			
**Protocol & registration**	5	Indicate whether a review protocol exists and where it can be accessed; and, if available, provide registration information, including registration number.	Materials and Methods/ 1st paragrph
**Eligibility criteria**	6	Specify study characteristics (e.g., PICOS, length of follow-up) and report characteristics (e.g., years considered, language, publication status) used as criteria for eligibility, giving rationale. Clearly describe eligible treatments included in the treatment network, and note whether any have been clustered or merged into the same node (with justification).	Materials and Methods/ Inculusion and exculusion
**Information sources**	7	Describe all information sources (e.g., databases with dates of coverage, contact with study authors) in the search and date last searched.	Table S2
**Search**	8	Present full electronic search strategy for at least one database, including any limits used, such that it could be repeated.	Table S2
**Study selection**	9	State the process for selecting studies (i.e., screening, eligibility, included in systematic review, and, if applicable, included in the meta-analysis).	Materials and Methods / Study Identification / Inculusion and exculusion criteria
**Data collection**	10	Describe method of data extraction from reports (e.g., piloted forms, independently, in duplicate) and any processes for obtaining and confirming data.	Materials and Methods / Data extraction
**Data items**	11	List and define all variables for which data were sought (e.g., PICOS, funding sources) and any assumptions and simplifications made.	Materials and Methods / Data extraction and conversion
**Network geometry**	S1	Describe methods used to explore the geometry of the treatment network under study and potential biases related to it. This should include how the evidence base has been graphically summarized for presentation, and what characteristics were compiled and used to describe the evidence base to readers.	Materials and Methods / Modeling for network meta-analysis
**Risk of bias within**	12	Describe methods used for assessing risk of bias of individual studies (including specification of whether this was done at the study or outcome level), and how this information is to be used in any data synthesis.	Materials and Methods / Quality appraisal
**Summary measures**	13	State the principal summary measures (e.g., risk ratio, difference in means). Also describe the use of additional summary measures assessed, such as treatment rankings, as well as modified approaches used to present summary findings from meta-analyses.	Materials and Methods / Outcome
**Planned methods of analysis**	14	Describe the methods of handling data and combining results of studies for each network meta-analysis. This should include, but not be limited to: Handling of multi-arm trials; Selection of variance structure; Selection of prior distributions in Bayesian analyses; and Assessment of model fit.	Materials and Methods / Statistical analyses
**Assessment of inconsistency**	S2	Describe the statistical methods used to evaluate the agreement of direct and indirect evidence in the treatment network(s) studied. Describe efforts taken to address its presence when found.	Materials and Methods / Statistical analyses
**Risk of bias across**	15	Specify any assessment of risk of bias that may affect the cumulative evidence.	Materials and Methods / Publication bias
**Additional analyses**	16	Describe methods of additional analyses if done, indicating which were pre-specified. This may include, but not be limited to, the following: Sensitivity or subgroup analyses; Meta-regression analyses; Alternative formulations of the treatment network; and Use of alternative prior distributions for Bayesian analyses (if applicable).	Materials and Methods / Sensitivity analyses
**Results**			
**Study selection**	17	Give numbers of studies screened, assessed for eligibility, and included in the review, with reasons for exclusions at each stage, ideally with a flow diagram.	Results / Study identification Figure 1, Table S2, Table S3
**Network structure**	S3	Provide a network graph of the included studies to enable visualization of the geometry of the treatment network.	Fugre 2
**Network geometry**	S4	Provide a brief overview of characteristics of the treatment network. This may include commentary on the abundance of trials and randomized patients for the different interventions and pairwise comparisons in the network, gaps of evidence in the treatment network, and potential biases reflected by the network structure.	Results / Network model formation / Figure 2
**Study characteristics**	18	For each study, present characteristics for which data were extracted (e.g., study size, PICOS, follow-up period) and provide the citations.	Table 1
**Risk of bias within**	19	Present data on risk of bias of each study and, if available, any outcome level assessment.	Table S4, Figure S1, Methodological quality
**Results of individual studies**	20	For all outcomes considered (benefits or harms), present, for each study: (1) simple summary data for each intervention group, and (2) effect estimates and confidence intervals. Modified approaches maybe needed to deal with information from larger networks.	Table 1

**Synthesis of results**	21	Present results of each meta-analysis done, including confidence/credible intervals. In larger networks, authors may focus on comparisons versus a particular comparator (e.g. placebo or standard care), with full findings presented in an appendix. League tables and forest plots may be considered to summarize pairwise comparisons. If additional summary measures were explored (such as treatment rankings), these should also be presented.	Outcomes / Figure 3, Figure 4, Figure S2, Figure S3, Table 2
**Exploration for inconsistency**	S5	Describe results from investigations of inconsistency. This may include such information as measures of model fit to compare consistency and inconsistency models, P values from statistical tests, or summary of inconsistency estimates from different parts of the treatment network.	Inconsistency test Table S5, Table S6
**Risk of bias across**	22	Present results of any assessment of risk of bias across studies for the evidence base being studied.	Publicaton bias, Figure S6
**Additional analyses**	23	Give results of additional analyses, if done (e.g., sensitivity or subgroup analyses, meta- regression analyses, alternative network geometries studied, alternative choice of prior distributions for Bayesian analyses, and so forth).	Sensitivity analysis / Figure S4, Figre S5
**Discussion**			
**Summary of evidence**	24	Summarize the main findings, including the strength of evidence for each main outcome; consider their relevance to key groups.	Discussion / Findings and implications
**Limitations**	25	Discuss limitations at study and outcome level (e.g., risk of bias), and at review level (e.g., incomplete retrieval of identified research, reporting bias). Comment on the validity of the assumptions, such as transitivity and consistency. Comment on any concerns regarding network geometry (e.g., avoidance of certain comparisons).	Discussion Linitations
**Conclusions**	26	Provide a general interpretation of the results in the context of other evidence, and implications for future research.	Conclusion
**Funding**	27	Describe sources of funding for the systematic review and other support (e.g., supply of data); role of funders for the systematic review. This should also include information regarding whether funding has been received from manufacturers of treatments in the network and/or whether some of the authors are content experts with professional conflicts of interest that could affect use of treatments in the network.	Funding

Note. PICOS, population, intervention, comparators, outcomes, study design.

Table S2: Keywords and search results in difference databases

Database	Keyword	Date	Results
Scopus	"TITLE-ABS-KEY(""polycystic ovary syndrome"" OR PCOS OR ""polycystic ovarian syndrome"" OR ""polycystic ovaries"") AND TITLE-ABS-KEY(exercise* OR ""physical fitness"" OR ""physical activity"" OR aerobic OR ""working out"" OR running OR cardio) AND TITLE-ABS-KEY(rct OR ""randomized control trial"" OR ""randomized controlled trial"" OR ""controlled trial"" OR cohort OR ""case control"")"	2025.03.16	531
Web of Science	TS = ("polycystic ovary syndrome" OR PCOS OR "polycystic ovarian syndrome" OR "polycystic ovaries") AND TS = (exercise* OR "physical fitness" OR "physical activity" OR aerobic OR "working out" OR running OR cardio) AND TS = (rct OR "randomized control trial" OR "randomized controlled trial" OR "controlled trial" OR cohort OR "case control")	2025.03.16	476
Cochrane CENTRAL	(polycystic ovary syndrome or pcos or polycystic ovarian syndrome or polycystic ovaries):ti,ab,kw AND (exercise or physical fitness or physical activity or aerobic or working out or running or cardio resistance training or high intensity interval training or HIIT or yoga or tai chi or pilates or swimming):ti,ab,kw AND (rct or randomized control trial or randomized controlled trial or controlled trial or cohort or case control):ti,ab,kw	2025.03.16	349
EBSCO	(MH "Polycystic Ovary Syndrome" OR TI ("polycystic ovary syndrome" OR PCOS OR "hyperandrogenism" OR "oligoovulation" OR "anovulation" OR "menstrual irregularity") OR AB ("polycystic ovary syndrome" OR PCOS)) AND (MH "Exercise" OR MH "Exercise Therapy" OR TI (exercise* OR train* OR "physical activit*" OR aerobic* OR "resistance train*" OR yoga OR pilates OR HIIT OR "high-intensity interval training" OR MICT OR "moderate-intensity continuous training" OR "lifestyle intervention" OR "circuit training" OR "combined exercise" OR "mind-body exercise" OR "behavioral modification") OR AB (exercise* OR train*)) AND (MH "Randomized Controlled Trials" OR PT "randomized controlled trial" OR TI ("randomized controlled trial" OR RCT OR "randomly assigned" OR "random allocation" OR "clinical trial") OR AB ("randomized controlled trial" OR RCT))	2025.03.16	190
MEDLINE	(TI ("polycystic ovary syndrome" OR PCOS OR "polycystic ovarian syndrome" OR "polycystic ovaries") OR AB ("polycystic ovary syndrome" OR PCOS)) AND (MH "Exercise" OR MH "Exercise Therapy" OR TI (exercise* OR "physical fitness" OR "physical activity" OR aerobic OR "working out" OR running OR cardio) OR AB (exercise* OR "physical activity")) AND (MH "Randomized Controlled Trials" OR PT "randomized controlled trial" OR TI (rct OR "randomized control trial" OR "randomized controlled trial" OR "controlled trial") OR AB (rct OR "randomized controlled trial"))	2025.03.16	167
Pubmed	(("Polycystic Ovary Syndrome"[Mesh] OR "Polycystic Ovary Syndrome" OR PCOS OR "Stein-Leventhal Syndrome" OR "Hyperandrogenism" OR "Oligoovulation" OR "Anovulation") AND ("Exercise"[Mesh] OR "Exercise Therapy"[Mesh] OR "Exercise Movement Techniques"[Mesh] OR "Aerobic Exercise"[Mesh] OR "Resistance Training"[Mesh] OR "High-Intensity Interval Training" OR "Yoga"[Mesh] OR "Tai Ji"[Mesh] OR	2025.03.16	117

"Pilates" OR "Swimming" OR "Cycling" OR "Physical Exertion" OR "Physical Fitness" OR "Exercise Training" OR "Workout" OR "HIIT")) AND ("Randomized Controlled Trial"[Publication Type] OR RCT OR "randomized controlled trial" OR "randomly allocated" OR "random allocation" OR "placebo-controlled trial" OR "controlled clinical trial"[ptyp])

Table S3: Studies excluded from the analysis along with the reasons for their exclusion

Authors & year	Title	Exclusion reasons
**Zhao et al., 2022**	Comparative effectiveness of aerobic exercise versus Yi Jin Jing on ovarian function in young overweight/obese women with polycystic ovary syndrome: study protocol for a randomized controlled trial	Protocol
**Santos et al., 2021 [18]**	Effect of high-intensity interval training on metabolic parameters in women with polycystic ovary syndrome: A systematic review and meta-analysis of randomized controlled trials	Review of literature
**Stener-Victorin, et al. 2012**	Effects of acupuncture and exercise on insulin sensitivity, adipose tissue characteristics, and markers of coagulation and fibrinolysis in women with polycystic ovary syndrome: secondary analyses of a randomized controlled trial	No relevant outcome data
**Richards et al., 2021 [59]**	HIIT'ing or MISS'ing the Optimal Management of Polycystic Ovary Syndrome: A Systematic Review and Meta-Analysis of High- Versus Moderate-Intensity Exercise Prescription	Review of literature
**Jedel et al., 2011**	Impact of electro-acupuncture and physical exercise on hyperandrogenism and oligo/amenorrhea in women with polycystic ovary syndrome: a randomized controlled trial	No relevant outcome data
**Kiel et al., 2020**	Improving reproductive function in women with polycystic ovary syndrome with high-intensity interval training (IMPROV-IT): study protocol for a two-centre, three-armed randomised controlled trial	No relevant outcome data
**Ryan et al., 2020**	Moderate-Intensity Exercise and High-Intensity Interval Training Affect Insulin Sensitivity Similarly in Obese Adults	Unsuitable population
**Ramos et al., 2016**	Quality of Life in Women with Polycystic Ovary Syndrome after a Program of Resistance Exercise Training	Not RCT
**Kogure et al., 2016**	Resistance Exercise Impacts Lean Muscle Mass in Women with Polycystic Ovary Syndrome	Unsuitable population
**Woodward et al., 2020**	Supervised exercise training and increased physical activity to reduce cardiovascular disease risk in women with polycystic ovary syndrome: study protocol for a randomized controlled feasibility trial	Protocol
**Li et al., 2018**	Tai chi for overweight/obese adolescent and young women with polycystic ovary syndrome: study protocol for a randomized controlled trial	Protocol
**Nasiri et al., 2022**	The Effect of High Intensity Intermittent and Combined (Resistant and Endurance) Trainings on Some Anthropometric Indices and Aerobic Performance in Women with Polycystic Ovary Syndrome: A Randomized Controlled Clinical Trial Study	No relevant outcome data
**Hiam et al., 2019**	The effectiveness of high intensity intermittent training on metabolic, reproductive and mental health in women with polycystic ovary syndrome: study protocol for the iHIT- randomised controlled trial	Protocol
**Laredo et al., 2004**	Effect of exercise in women with polycystic ovary syndrome (PCOS): A randomized controlled trial of diet and exercise compared with diet alone	Irrelevant intervention
**Bruner et al., 2006**	Effects of exercise and nutritional counseling in women with polycystic ovary syndrome	Irrelevant intervention
**Orio et al., 2016**	Oral contraceptives versus physical exercise on cardiovascular and metabolic risk factors in women with polycystic ovary syndrome: a randomized controlled trial	Irrelevant intervention
**Nybacka et al., 2011**	Randomized comparison of the influence of dietary management and/or physical exercise on ovarian function and metabolic parameters in overweight women with polycystic ovary syndrome	Irrelevant intervention
**Ribeiro et al., 2021**	Short-Term Aerobic Exercise Did Not Change Telomere Length While It Reduced Testosterone Levels and Obesity Indexes in PCOS: A Randomized Controlled Clinical Trial Study	No relevant outcome data
**Saremi et al., 2016**	Effect of resistance exercises with calcium consumption on level of anti-mullerian hormone and some metabolic indices in women with polycystic ovarian syndrome	Irrelevant intervention
**Verma et al., 2023**	Effect of Yoga Therapy on Health Outcomes in Women With Polycystic Ovary Syndrome: A Systematic Review and Meta-Analysis	Review of literature
**Stener-Victorin et al., 2009**	Effects of acupuncture and exercise on insulin sensitivity, adipose tissue characteristics, and markers of coagulation and fibrinolysis in women with polycystic ovary syndrome: secondary analyses of a randomized controlled trial	Irrelevant intervention

Table S4: A list of basic characteristics of the studies included in the meta-analysis [15,34–51]

First Author, year	Country	Sample size (n)	Age range (mean ± SD) year	BMI (mean ± SD) (kg.m−2)	Intervention	Control	Intervention duration (week)	Co-intervention	Outcome and Measurement Tool
**Turan et al., 2015 [34]**	Dokuz	CT = 14; CG = 16	24.45 ± 2.8	CT: 21.8±1.0; CG: 21.9±1.1	50–60 min per time, 3 times per week. 5-min warm-up, followed by a 5-min cool down; raised to a height of 15–20 min aerobic exercise at 65–70% of the maximum heart rate; Resistance exercise : 15 repetitions of each targeted muscle exercise, with a minimum resting period of 30s and a maximum of 1 min between each repetition, maintain an exercise intensity close to 5 or 6	not participate in a structured exercise program	8	general dietary and behavioral advice	HOMA-IR; total testosterone
**Philbois et al., 2022 [35]**	Brazil	MICT = 25; HIIT = 25; CG = 25	MICT:29 ± 5; HIIT:29 ± 4; CG: 29 ± 5	MICT:27.7 ± 5.7; HIIT:27.8 ± 4.2; CG: 29.2 ± 5.4	MICT: 1hour per time, 3 times per week. 5-min warm-up(50%–65% of reserve HR);50-min training (70%–80% of reserve HR); 5-min cool-down(40%–50% of reserve HR). HIIT: 35–45 min per time,3 times per week. 5-min warm-up(50%–65% of reserve HR);2-min HIIT corresponded to the sum of HR at rest and 85%–90% of the reserve HR; 3-min of intensity corresponding to the sum of HR at rest and 65%–70% of the reserve HR; 5-min cool-down(40%–50% of reserve HR).	without any exercise training	16	null	HOMA-IR; total testosterone
**Ribeiro et al., 2021 [36]**	Brazil	MICT = 28; HIIT = 29; CG = 30	MICT:29.14 ± 5.26; HIIT:28.97 ± 4.32; CG:28.50 ± 5.76	MICT:28.4 ±5.6; HIIT:28.7 ±4.8; CG:29.1 ±5.2	MICT: from 30min raisedto 50min per time, 3 times per week. 5-min warm-up(50%–60% of reserve HR);the target intensity areas of training followed the recommendations of ACSM; 5 min cool-down(50%–60% of reserve HR). HIIT: from 30min raisedto 50min per time,3 times per week. 5-min warm-up(50%–60% of reserve HR);The target intensity areas of training followed the recommendations of ACSM; 5-min cool down(50%–60% of reserve HR).	without any exercise training	16	maintain the same daily diet	Fasting insulin; Fasting blood glucose; total testosterone
**Nidhi et al., 2012 [37]**	India	Yoga = 35; MICT = 37	Yoga: 16.22 ± 1.13; MICT: 16.22 ± 0.93	Yoga: 20.30 ± 1.92; MICT: 21.22 ± 2.99	Yoga: 1hour per time, 7 times per week. consisted of asanas (yoga postures), pranayama, relaxation techniques, and meditation.	MICT: 1hour per time, 7 times per week. consisted of a set of physical movements and safe non-yogic breathing followed by supine rest (without instructions)	12	null	HOMA-IR
**Mohammadi et al., 2023 [38]**	Iran	HIIT = 14; CG = 14	HIIT: 24.2 ± 4.8; CG: 22.9 ± 5.3	HIIT: 29.5±4.5; CG: 31.4±2.6	HIIT: 3 times per week.a warm-up for each session (including 15 min of standard warm-up), a low-intensity run (50% of maximum aerobic speed) and then 3 repetitions of 30-s sprint running followed by 30 s of slow running and 5 min of dynamic stretching. The number of sets and laps for the HIIT program increased in the following weeks	without any exercise training	8	null	HOMA-IR; total testosterone
**Almenning et al., 2015 [39]**	Norwegian	HIIT = 8; RT = 8; CG = 9	27.2 ± 5.5	RT: 27.4 ± 6.9; HIIT: 26.1±6.5; CG: 26.5±5	HIIT: 3 times per week. four times 4 minutes HIT at 90–95% Hrmax, separated by 3 minutes of moderate intensity exercise at 70% of Hrmax;and one weekly session of ten times one minute with	recommended ≥150 minutes of weekly moderate-intensity	10	null	Fasting insulin; Fasting blood glucose; total testosterone

					maximal intensity HIT, separated by 1 minute of rest/very low activity. RT: consisted of eight dynamic strength drills with a resistance of 75% of one repetition maximum (1RM), with ten repetitions and three sets separated by one-minute rest between sets.	exercise without any follow-up			
**Benham et al., 2021 [15]**	Canada	MICT = 14; HIIT = 16; CG = 17	29.2± 4.7	31.4±8.4	MICT: 3 times per week. 5-min warm-up; 40 min of moderate-intensity aerobic exercise (50%–60% HRR, or 4-6/10 on a modified Borg scale); 5-min cool-down. HIIT: 3 times per week. 5-min warm-up; 10 cycles of 30 s at high-intensity (90% of heart rate reserve (HRR), or 9/10 on a modified Borg scale18) alternating with 90 s of low-intensity aerobic exercise; 5-min cool-down.	maintain usual level of physical activity	26	null	Fasting insulin; Fasting blood glucose
**Giallauria et al., 2008 [40]**	Italy	MICT = 62; CG = 62	MICT:22.8 ± 3.7; CG:22.6 ± 3.0	MICT:29.2 ± 2.9; CG:29.5 ± 3.5	MICT: 3 times per week. 5-min warm-up; 30 min on a bicycle ergometer with the target of 60–70% of the VO2; 5-min cool-down.	without any exercise training	12	null	Fasting insulin; Fasting blood glucose; total testosterone
**Patten et al., 2022 [41]**	Australia	MICT = 14; HIIT = 15	HIIT: 29.7 ± 4.8; MICT: 32.5 ± 6.2	MICT:29.2 ± 2.9; CG:29.5 ± 3.5	MICT: 3 times per week. 5-min warm-up(60–65% HRpeak); 45 min of continuous cycling at 60–75% HRpeak; 5-min cool-down(60–65% HRpeak). HIIT: 3 times per week. 5-min warm-up(60–65% HRpeak); twice weekly sessions of 12, 1 min intervals(90–100%HRpeak), separated by 1 min of active recovery at a light load and one weekly session of eight, 4 min intervals at 90–95% HRpeak, separated by a 2 min light load, activity recovery; 5-min cool-down(60–65% HRpeak).	null	12	null	Fasting insulin; Fasting blood glucose; total testosterone
**Nasiri et al., 2025 [42]**	Iran	CT = 15; CG = 15	23.8 ± 5.3	30.3 ± 3.9	CT: started at 30 min/50% intensity in Week 1 and increased weekly reach to 40 min/70% by Week 8. after resistance session performed endurance training: 24 total treadmill running sessions at 60-70% of their Target Heart Rate (THR).	only do normal daily accomplish ments and avoid any sports activities	8	null	HOMA-IR; total testosterone
**Patel et al., 2020 [43]**	America	Yoga = 13; CG = 9	Yoga: 30.9 ± 1.2; CG: 31.2 ± 2.3	31.2 ±2.3	Yoga: 1hour per time, 3 times per week. began with body awareness; practice included pranayama exercises (3-part yogic breath: ujjayi breath, alternate nostril breathing, and breath of fire), vinyasa flow yoga, restorative yoga asanas, imagery of healing energy and mindful "I am" statements.	don't change any aspects of their daily routine	12	null	Fasting insulin; Fasting blood glucose; total testosterone
**Furtado et al., 2024 [44]**	Brazil	RT = 30; MICT = 26	RT: 30.9 ± 1.2; MICT: 31.2 ± 2.3	RT: 28.45 ± 6; MICT: 28.29 ± 5.73	MICT: from 30min raisedto 50min per time, 3 times per week. carried out on a treadmill. Light (50%-64% HRmax), moderate (64%-77% HRmax), and vigorous (77%-94% HRmax) intensities were considered to calculate the progression. RT: 50-minute to one-hour per time,3 times per week.decreasing volume and increasing intensity, included bench press, leg extension, front lat pull-down, leg curl, lateral raise, leg press, triceps pulley, calf leg press, arm curl, and abdominal exercise.	null	16	null	Fasting insulin; Fasting blood glucose; total testosterone
**Vasheghani-Farahani et al., 2017 [45]**	Iran	CT = 16; CG = 14	CT: 27.7 ± 4.2; CG: 29 ± 5.39	CT: 28.8±5.88; CG: 25.7±2.15	CT: 3 times per week. Chair push up and Single Arm Row. Seated lower leg lift, Seated Straight leg lift, Stair Step and Chair Squat	resume their normal activities	12	null	HOMA-IR; total testosterone

					exercises were considered. 10 times per each bout				
**Aktas et al., 2022 [46]**	Turkey	MICT = 10; CG = 10	HIIT:25.1 ± 4.6; MICT: 24.6 ± 6.7	HIIT:28.7 ±6.9; MICT: 26.57±5.5	HIIT: 1hour per time, 3 times per week, Running (2minutes) and Walking (2 minutes)(Consecutive). MICT: 1hour per time, 3 times per week, Only Running (moderate tempo at a constant speed)	null	12	null	Fasting insulin; Fasting blood glucose
**Vizza et al., 2016 [47]**	Sydney	RT = 8; CG = 7	RT: 26 ± 7; CG: 29 ± 3	RT: 41.3 ±12.5; CG: 34±9.4	RT: 4 times per week, two supervised training sessions per week, 1hour per time, two home-based (unsupervised) exercise sessions consisting of lower-intensity calisthenics	continue with their current lifestyle	12	null	Fasting insulin; Fasting blood glucose; total testosterone
**Wang et al., 2024 [48]**	America	HIIT = 9; MICT = 11	MICT: 30.1 ± 6.3; HIIT: 32.4 ± 5.3	MICT: 32.6 ± 7.5; HIIT: 33.7 ± 6.3	15–30 minutes per day, 75 minutes of vigorous activity or 150 minutes of moderate activity per week.	null	8	null	Fasting insulin; Fasting blood glucose; total testosterone
**Nid hi et al., 2013 [49]**	India	Yoga = 37; MICT = 35	Yoga: 16.22 ± 1.13; CG: 16.22 ± 0.93	Yoga: 20.30 ± 1.92; MICT: 21.22 ± 2.99	Yoga: 1 hour daily, 7 days a week for 12 weeks (total 90 sessions), consisted of lecture (5 minutes) followed by physical practices (40 minutes), pranaya-ma (breathing exercise) (5 minutes), and relaxation (10minutes)	MICT: 1hour per time, 7 times per week. consisted of a set of physical movements and safe non-yogic breathing followed by supine rest (without instructions)	12	null	total testosterone
**Babaei Bonab et al., 2023 [50]**	Iran	MICT = 20; CG = 20	MICT: 16.01 ± 54.64 CG: 16.02 ± 32.47	MICT: 28.16 ± 0.8; CG: 28.57 ± 0.07	MICT: 3 times per week. 10-min warm-up; 30 minutes of main aerobic workout; 10-min cool-down.	continue with their current lifestyle	12	null	total testosterone
**Lopes et al., 2018 [51]**	Brazil	MICT = 23; HIIT = 22; CG = 24	30.2 ± 5.1	MICT: 29.3±5.6; HIIT: 29.0±4.8; CG: 29.9±5.3	MICT: 3 times per week. 5-min warm-up(50%–65% of reserve HR);30-50-min training (50–60%HRmax); 5-min cool-down(50%–65%HRmax). HIIT: 3 times per week. 5-min warm-up(50%–65%HRmax); 30-50-min training, 2-min HIIT 85%–90%HRmax; 3-min of intensity corresponding to 65%–70%HRmax; 5-min cool-down(50%–65%HRmax)	null	16	null	total testosterone

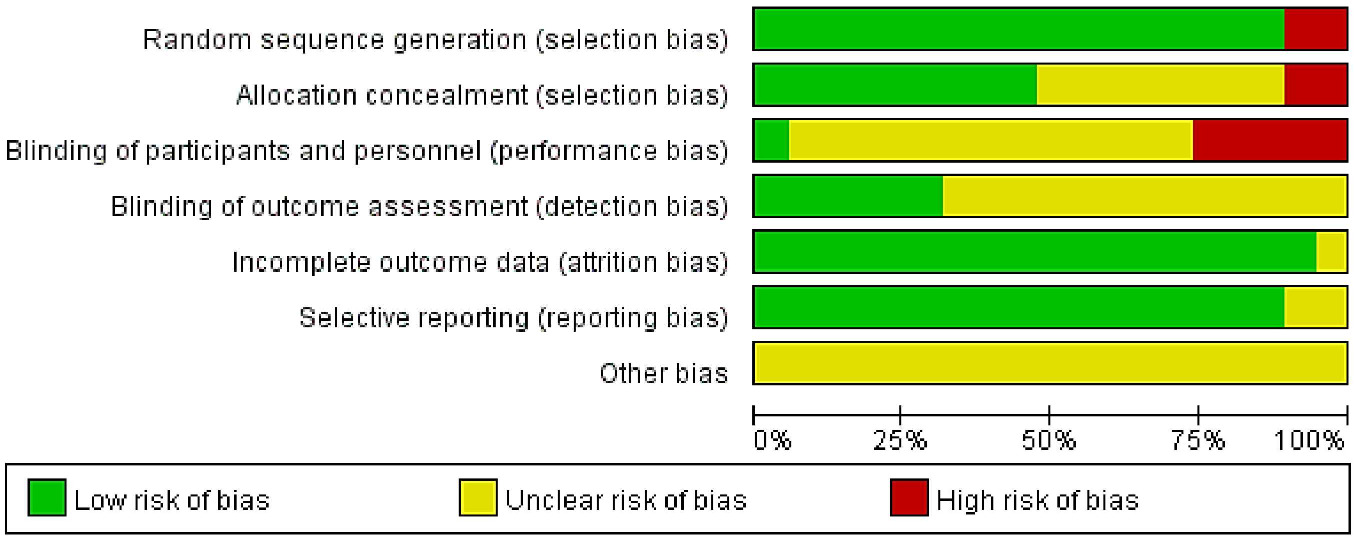

Figure S1: Summary of the quality assessment for included studies

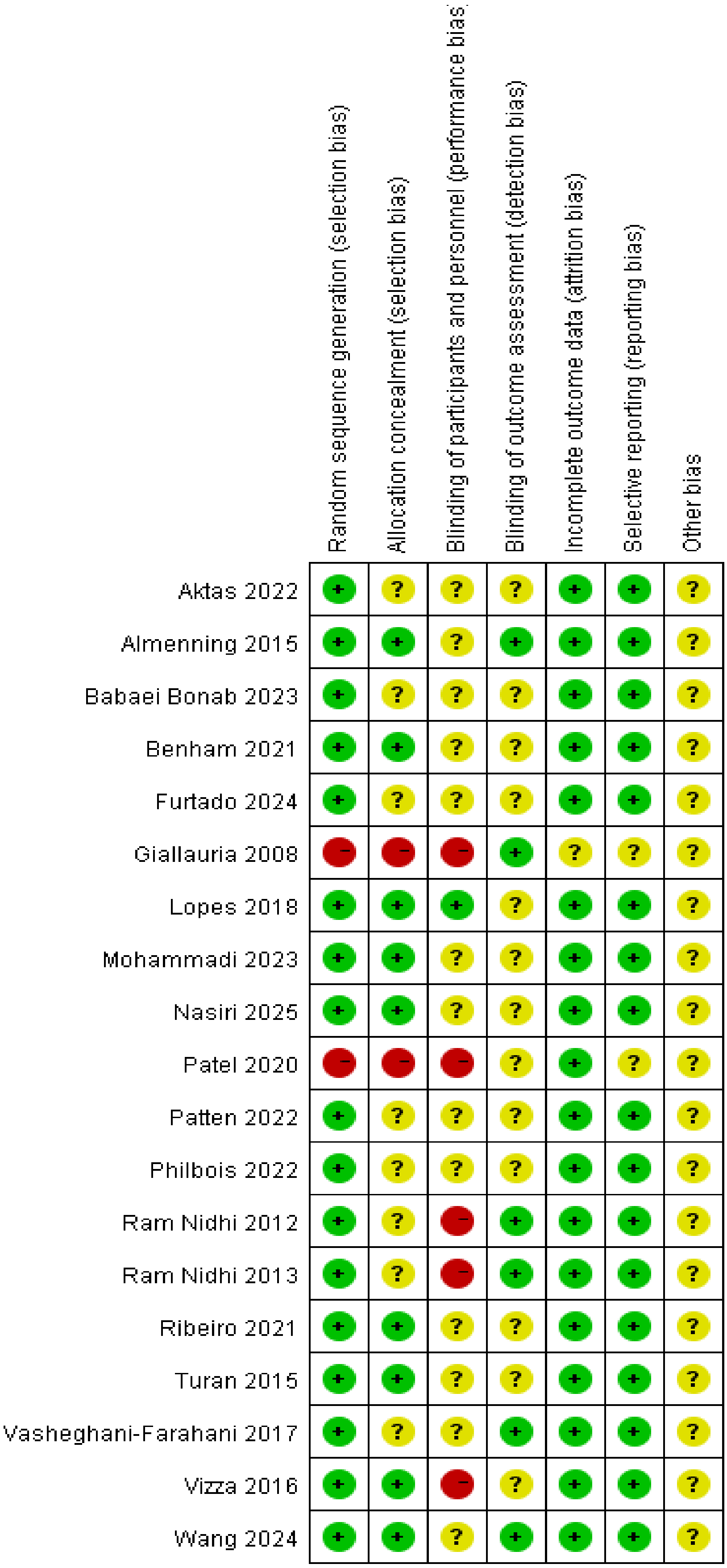

Figure S2: Risk of bias graph

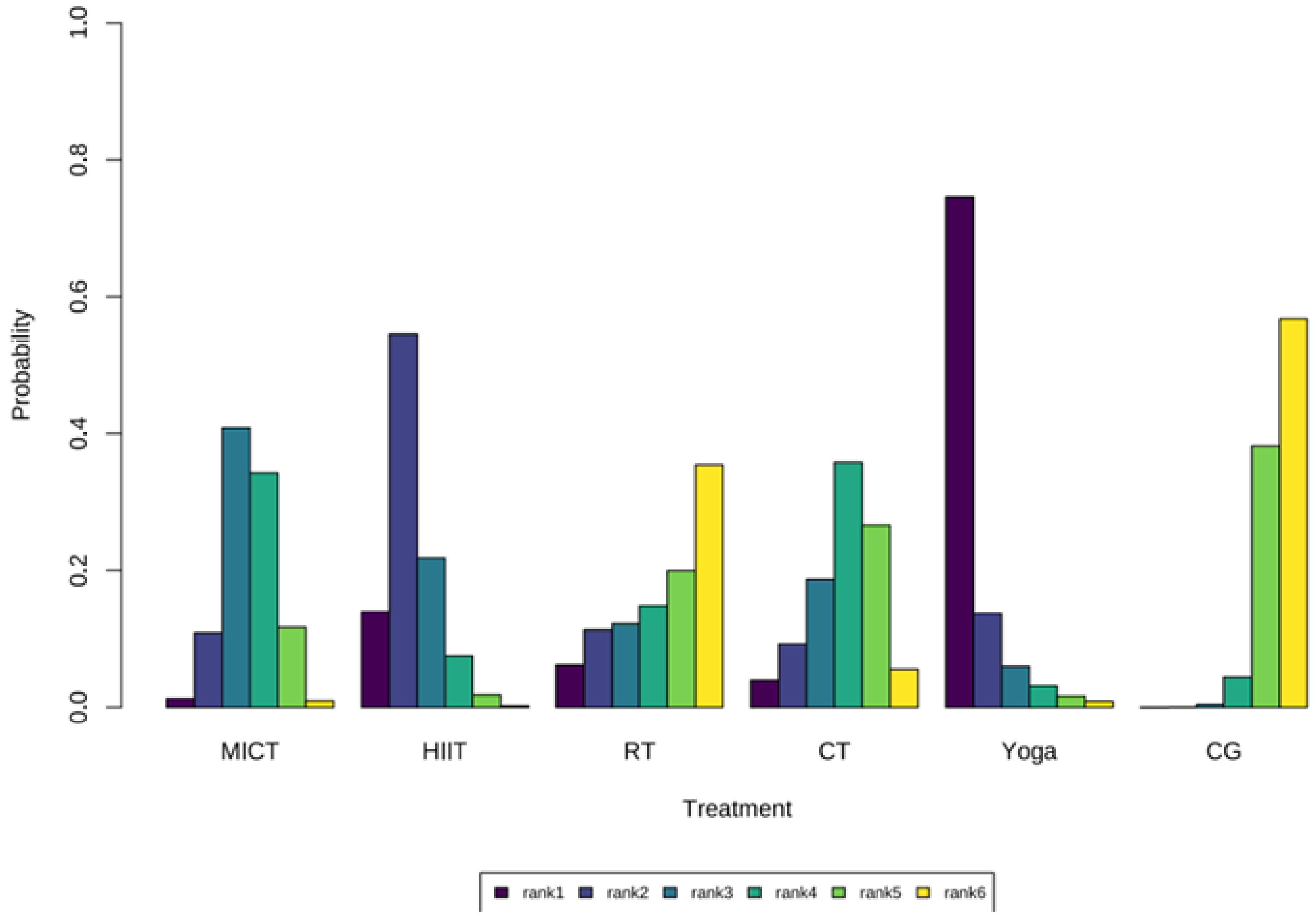

Figure S3: Probability ranking diagram of different exercise interventions aimed at improving HOMA-IR in women with PCOS.

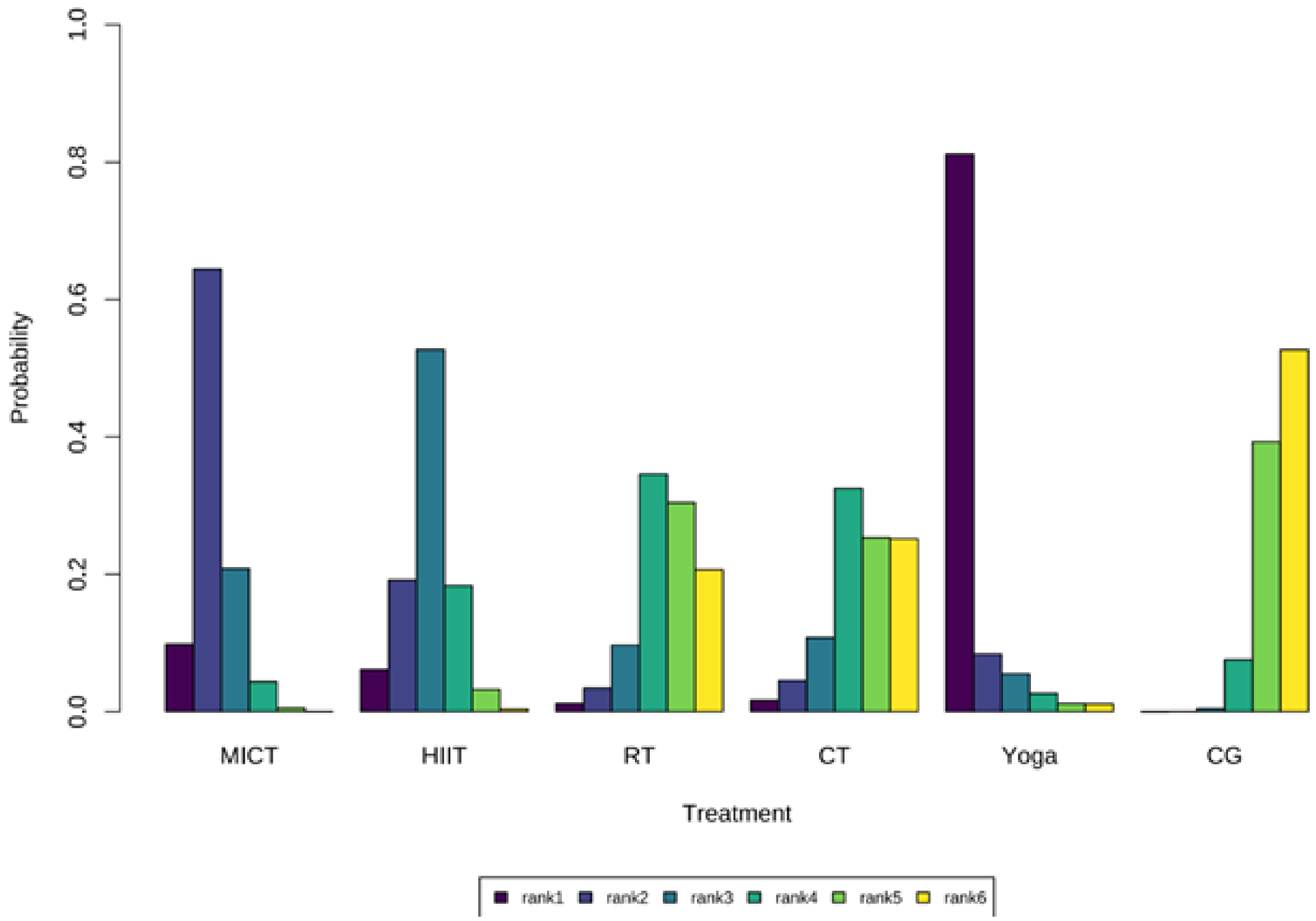

Figure S4: Probability ranking diagram of different exercise interventions aimed at reducing total testosterone in women with PCOS.

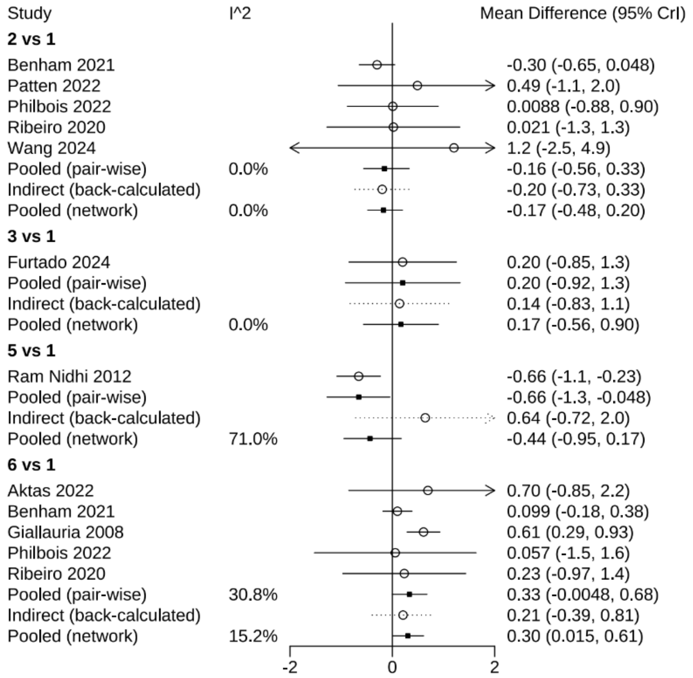

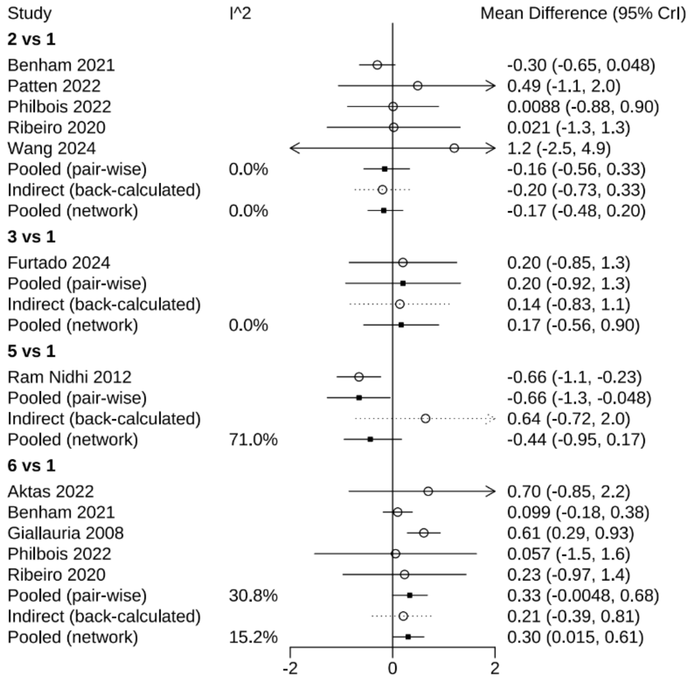

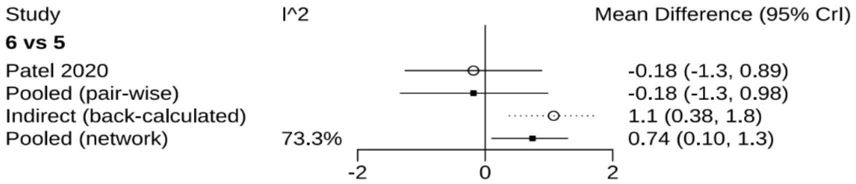

Figure S5: Individual study results (with studies excluded) on HOMA-IR grouped by treatment comparison**.**

Note: 1 = Moderate intensity continuous aerobic training; 2 = High intensity interval training; 3 = Resistance train; 4 = concurrent training; 5 = Yoga; 6 = Control group

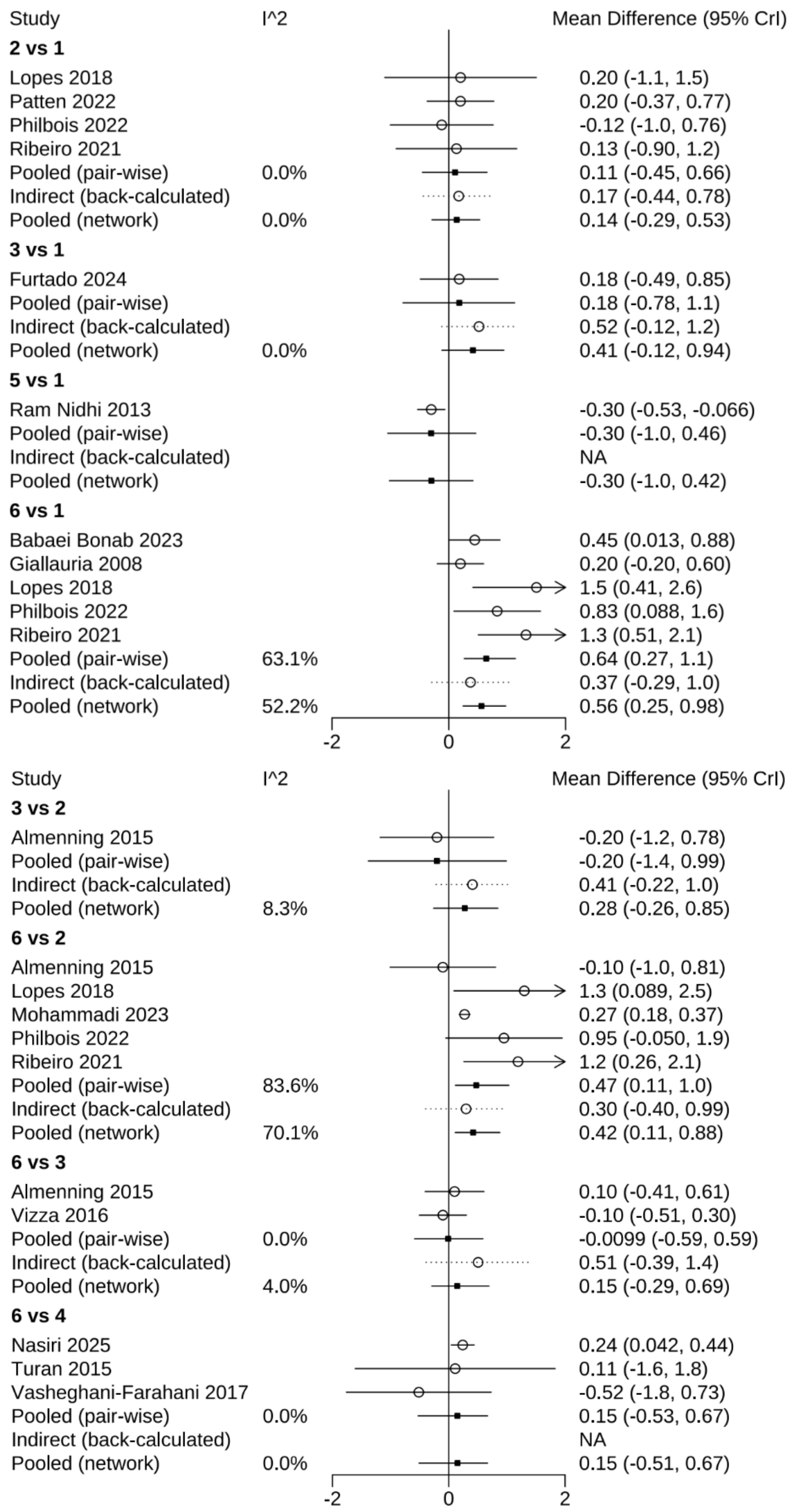

Figure S6: Individual study results (with studies excluded) on total testosterone grouped by treatment comparison**.**

Note: 1 = Moderate intensity continuous aerobic training; 2 = High intensity interval training; 3 = Resistance train; 4 = concurrent training; 5 = Yoga; 6 = Control group.

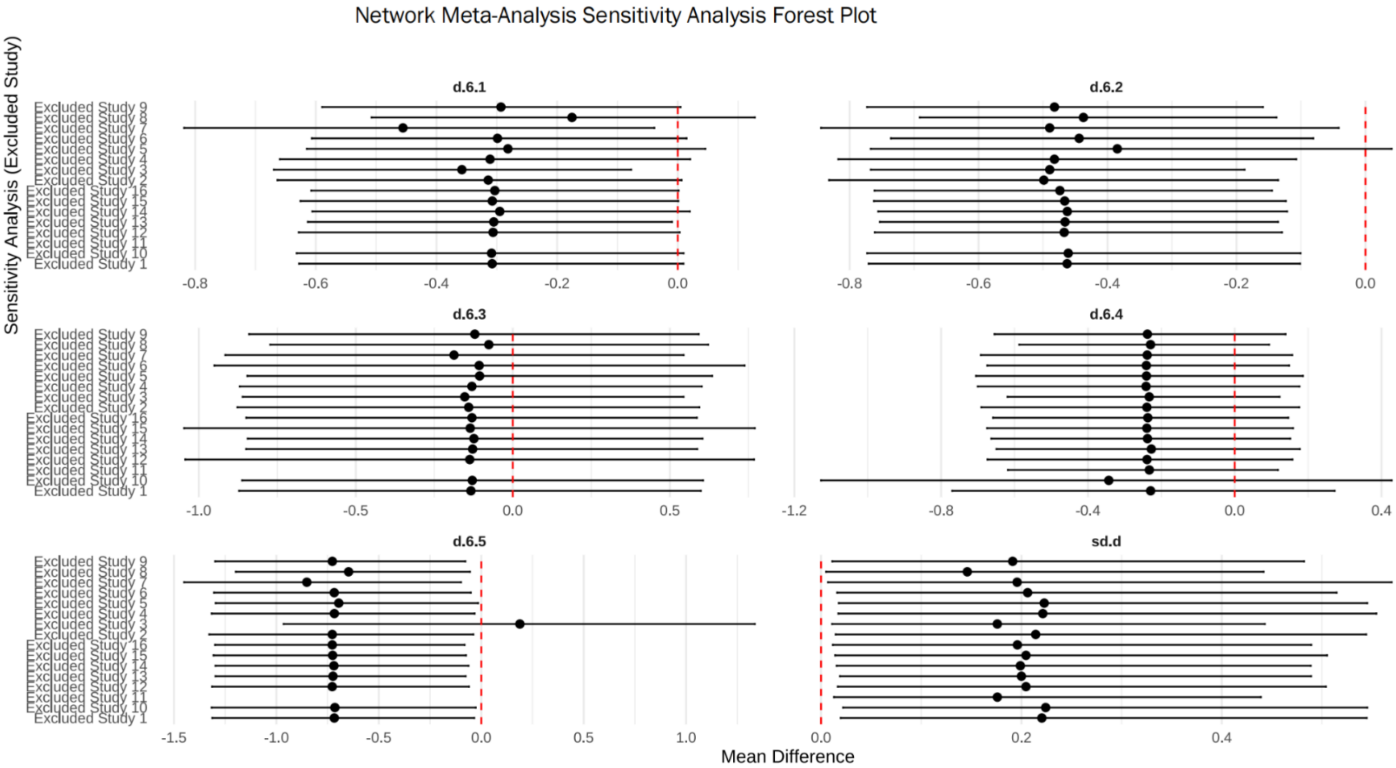

Figure S7: The forest plots display the HOMA-IR results of the sensitivity analysis.

Note: Study 1: Turan 2015; Study 2: Philbois 2022; Study 3: Ram Nidhi 2012; Study 4: Ribeiro 2020; Study 5: Mohammadi 2023; Study 6: Almenning 2015; Study 7: Benham 2021; Study 8: Giallauria 2008; Study 9: Patten 2022 Study 10: Nasiri 2025; Study 11: Patel 2020; Study 12: Furtado 2024; Study 13: Vasheghani-Farahani 2017; Study 14: Aktas 2022; Study 15: Vizza 2016; Study 16: Wang 2024.

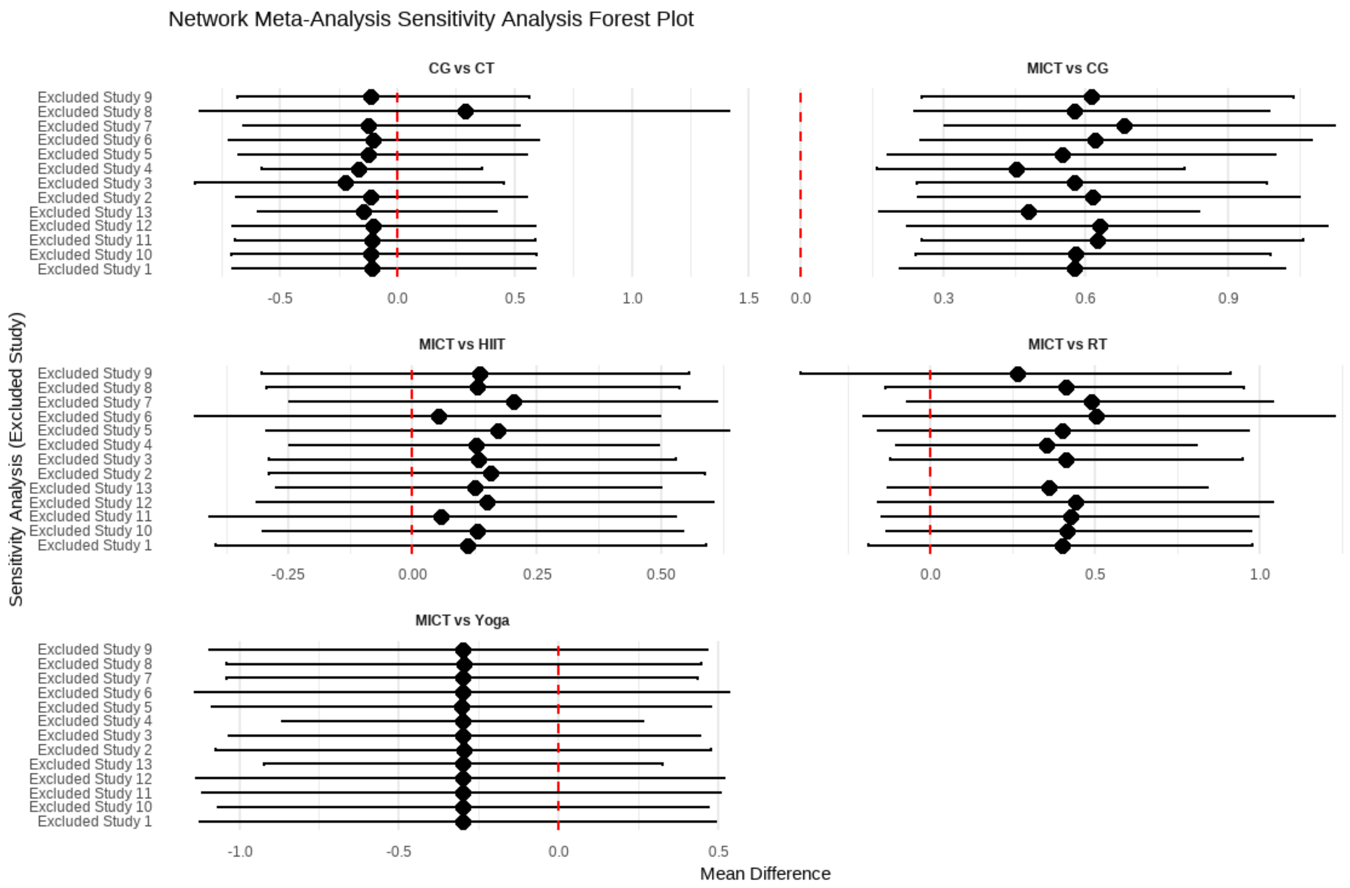

FigureS8: The forest plots display the total testosterone results of the sensitivity analysis**.**

Note: Study 1: Patten 2022; Study 2: Furtado 2024; Study 3: Vasheghani-Farahani 2017; Study 4: Ram Nidhi 2013; Study 5: Ribeiro 2020; Study 6: Philbois 2022; Study 7: Almenning 2015; Study 8: Giallauria 2008; Study 9: Nasiri 2025 Study 10: Vizza 2016; Study 11: Turan 2015; Study 12: Mohammadi 2023; Study 13: Babaei Bonab 2023.

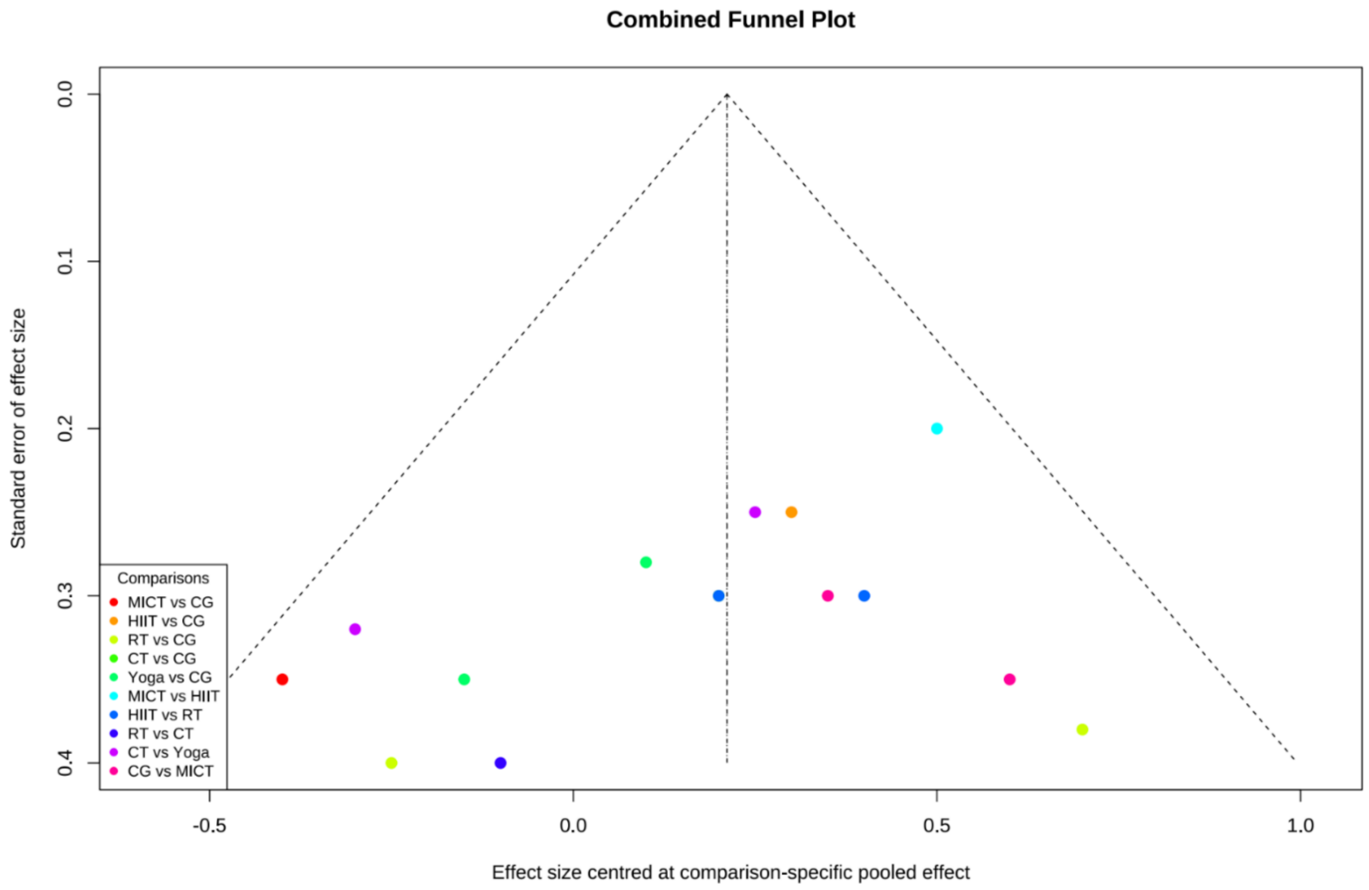

Figure S9: Publication bias funnel plots on HOMA-IR in women with PCOS.

Note: MICT = Moderate intensity continuous aerobic training; HIIT = High intensity interval training; RT = Resistance train; CT = concurrent training; Yoga = Yoga; CG = Control group.

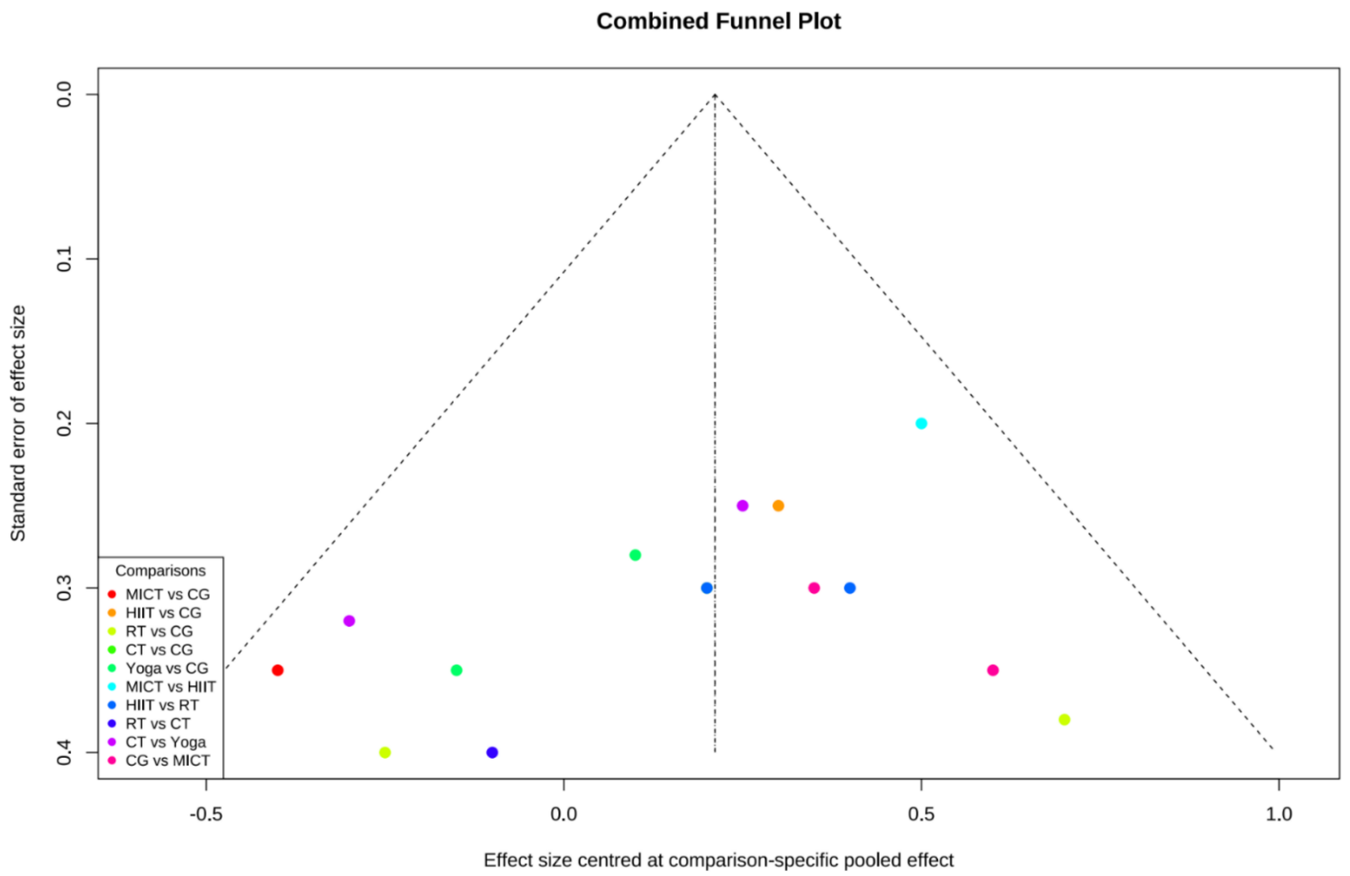

Figure S10: Publication bias funnel plots on total testosterone in women with PCOS.

Note: MICT = Moderate intensity continuous aerobic training; HIIT = High intensity interval training; RT = Resistance train; CT = concurrent training; Yoga = Yoga; CG = Control group
